# An Audit of Australian Bread with a Focus on Loaf Breads and Whole Grain

**DOI:** 10.3390/nu10081106

**Published:** 2018-08-16

**Authors:** Sara Grafenauer, Felicity Curtain

**Affiliations:** Grains & Legumes Nutrition Council, Sydney, NSW 2060, Australia; contactus@glnc.org.au

**Keywords:** bread, whole grain, wholemeal, dietary fibre, sodium, gluten free, carbohydrate, sugar, protein, health claim

## Abstract

Bread is a vehicle for a range of nutrients within the Australian diet, but has been the target of negative press. The aim of this study was to examine bread products, particularly white, whole grain and gluten-free loaves, including nutrients, health claims and Health Star Rating (HSR). An audit of four supermarkets and a bakery franchise (2017) was compared with 2014 data. Median and range was calculated for whole grain content, dietary fibre, sodium, protein, carbohydrate and sugar. Of all breads (*n* = 456), 29% were eligible to make a whole grain claim with 27% very high in whole grain (≥24 g/serve), an 18% increase from 2014. Within loaves (*n* = 243), 40% were at least a source of whole grain (≥8 g/serve), 79% were at least a source of dietary fibre, 54% met the sodium reformulation target (≤400 mg/100 g), 78% were a ‘source’ and 20% were a ‘good source’ of protein (10 g/serve), and 97% were low in sugar. Despite significant differences between loaves for all nutrients assessed, HSR did not differ between white and whole grain varieties. Compared to 2014, there were 20 fewer white loaves and 20 additional whole grain loaves which may assist more Australians achieve the 48 g whole grain daily target intake.

## 1. Introduction

Bread is a staple food in many countries around the world and is one of the key products within the cereal and cereal products group of foods. It is a useful food vehicle for fortification of at-risk micronutrients in the Australian diet, specifically serving as a strategy to help reduce the incidence of Wernicke-Korsakoff syndrome [1] (via thiamin) and improve folate [2] and iodine status [3,4]. The National Nutrition and Physical Activity Survey (NNPAS), as part of the Australian Health Survey 2011–2013 [5], identified that within the bread category, plain, regular bread and bread rolls are consumed by 66% of Australians with a median consumption of 72 g (approximately two average slices) on the day prior to the NNPAS interview [5]. Most often, this was white bread (58%) with mixed grain and wholemeal varieties accounting for 18% [5]. Since 1995, the amount of bread consumed has decreased by 20 g/person/day [6]. 

Grain foods, including bread products, have been shown to provide a range of shortfall nutrients in the U.S. [7,8]; and from NNPAS, this group of foods are known to supply B vitamins, iron, calcium, magnesium, selenium, zinc and dietary fibre for Australians [9]. Bread is also a potential source of whole grain in the diet. Meta-analyses and systematic reviews of all-cause and cause-specific mortality are supportive of whole grains [10,11,12] and analyses of randomised clinical trials provide additional support indicating that whole grains produce reductions in low density lipoprotein (LDL) cholesterol, total cholesterol and percentage body fat compared to diets without them, and show improvements in postprandial glucose levels and glucose homeostasis [13,14,15]. Furthermore, there is a growing understanding that whole grains are more than just fibre [16], providing a range of micronutrients and other bioactive compounds with a variety of functions.

The Grains & Legumes Nutrition Council (GLNC) led the development of the Code of Practice for Whole Grain Ingredient Content Claims (The Code) in Australia and New Zealand, and measures compliance with this code by conducting rolling category audits of grain foods. The Code, developed in 2013, provides guidance on the use of whole grain ingredient content claims with cut-off values of at least 8 g per manufacturer serve (contains whole grain), at least 16 g per serve (high in whole grain) and at least 24 g per serve (very high in whole grain) [17]. Food Standards Australia New Zealand (FSANZ) do not regulate claims describing the amount of whole grain in foods but define whole grain as the intact grain or the dehulled, ground, milled, cracked or flaked grain where the constituents—endosperm, germ and bran—are present in such proportions that represent the typical ratio of those fractions occurring in the whole cereal, and includes wholemeal [18]. The Code was designed to complement existing food standards and to assist consumers in meeting the 48 g Daily Target Intake (DTI). The whole grain DTI was determined by an expert round table [19,20] and is based on the scientific evidence that people who eat at least 48 grams of whole grain each day are less likely to develop coronary heart disease [11] and this is consistent with the target used in the U.S. [21].

From NNPAS, the median daily whole grain intake was calculated at 21 g for adults [22] with only 31% meeting the 48 g DTI. This low level of whole grain consumption is not uncommon [23,24] despite higher levels of consumption being related to lower risk for most non-communicable disease endpoints [25,26,27,28]. Therefore, there is need for clear labeling, marketing and increased awareness to assist individuals in making choices within this category, which may be associated with better health outcomes.

Despite bread’s role in the diet and in providing key nutrients, over the last two decades it has often been the focus of criticism, regarding the sodium, added sugar and overall carbohydrate content due to health concerns and popular diet trends like low carbohydrate and gluten-free. Many consumers hold beliefs that breads are ‘fattening’ and cause bloating [29]. In addition to this, the Health Star Rating (HSR), the current front-of-pack nutrition communication tool used in Australia, does not utilise whole grain content as part of the algorithm calculating the number of stars a product can display. This potentially impacts the consumers’ ability to differentiate between refined and whole grain products within this and other grain food categories.

The aim of this study was to examine the variety of bread products available, particularly loaves (white, whole grain and gluten free), including nutrients and health claims with a focus on whole grain content, dietary fibre, sodium, protein, carbohydrate and sugar and also the HSR. 

## 2. Materials and Methods 

An audit of bread was conducted using a recognised process for the collection of nutrient composition [30] from four major supermarkets in the Sydney Metropolitan area with the addition of a Bakery franchise (Bakers Delight^TM^, Camberwell, Victoria, Australia) during September 2017. Permission to visit each of the local supermarkets was sought prior to the audit process, and consent was requested from the store manager to proceed on the day of the audit (Woolworths, Neutral Bay; Independent Grocers of Australia (IGA), Cremorne; Coles, Neutral Bay; Aldi, North Sydney). Photographs were taken with smartphones of all available products within each bread category. Auditors captured all sides of the packets and ensured inclusion of any writing or logos including front-of-pack, nutrition information, the nutrition information panel (NIP) and health claims. Bread products included in this audit comprised of bread loaves; rolls; sandwich alternatives including wraps, thins, Lebanese and pita bread; as well as flatbreads such as Turkish, ciabatta, Indian, foccacia and yiros. Bakery breakfast products such as fruit bread, brioche, English muffins, crumpets and pancakes were also included. Loaf breads were categorised into three main types of bread loaves for analysis (white, wholemeal/whole grain and gluten-free options). Products excluded from this audit comprised of the store’s bakery goods section (in-store bakery), pizza bases, and bread sticks such as baguettes and breads containing added ingredients (e.g., olives and cheese). If products were sold out during the data collection period, auditors noted this and conducted an online search of the nutrition information on the manufacturer’s website. Product data from Bakers Delight^TM^ were taken from their website as there is no nutritional information or claims made on the packaging supplied to consumers. 

The information obtained from the photographs was entered into a Microsoft^®^ Excel^®^ spreadsheet (Version 2013, Redmond, Washington, USA). Information required for data entry included the NIP per serve and per 100 g, the percent of whole grains, ingredients and health-related claims (Table 1) including whole grain, dietary fibre, sodium, protein, carbohydrate and sugar and front-of-pack information (HSR). Additionally, the number of products eligible to make nutrition claims was documented. The eligibility of providing a whole grain content claim was assessed in line with The Code. The total number of products eligible for a claim was noted as well as the percentage of these that were eligible for each level (contains whole grain, high in whole grain and very high in whole grain) of claim. If any products registered or unregistered made a whole grain or nutrient claim that was not compliant with The Code or were deemed misleading, they were entered into a whole grain compliance monitoring document for follow up by GLNC.

Data were cross-checked with manufacturer data and following data entry, results were independently checked by a second reviewer for any inconsistencies or errors. Results were compared with data from the bread audit conducted by GLNC in 2014 [31] regarding change in sodium content and the number of whole grain products. This audit utilised the same methods and quality processes as the current audit, and collected a similar number of loaf breads (*n* = 242) and total breads (*n* = 474) from the same supermarket locations. 

### Statistics

All data were checked for normality using Shapiro–Wilks (IBM SPSS^®^, version 25.0, IBM Corp., Chicago, IL, USA). Kruskal-Wallis test (IBM SPSS^®^, version 25.0, IBM Corp., Chicago, IL, USA) was used to compare differences in whole grain content and nutrients in loaf breads. Descriptive data was used to present the products meeting claimable amounts for whole grain, dietary fibre, the reformulation target for sodium, protein, sugar and HSR. 

## 3. Results

Of 456 bread products in the total bread category, there were 243 loaves (80 white loaves, 141 whole grain loaves, 22 gluten-free loaves). The remaining bread category was made up of 53 rolls including burger buns and hot dog rolls (34 white rolls, 13 whole grain rolls, 6 gluten-free rolls), 73 sandwich alternatives (55 wraps, 3 sandwich thins, 11 Lebanese breads, 4 pita breads), 24 flatbreads (21 Turkish breads, 2 ciabatta breads, 1 Indian naan bread), and 63 bakery breakfast products (3 crumpets, 33 fruit breads, 4 English muffins, 10 pancakes & pikelets, 9 brioche, and 4 bagels). 

With the exception of protein, other nutrient data were not normally distributed. The whole grain content and key nutrients in loaf breads (white, whole grain and gluten-free) and in the total bread category are presented in Table 2 (median and range). A Kruskal-Wallis H test found a statistically significant difference between loaf breads in whole grain content (X^2^ = 13.385, *p* = 0.001), dietary fibre (X^2^ = 43.307, *p* < 0.0001), sodium (X^2^ = 19.019, *p* < 0.0001), protein (X^2^ = 52.748, *p* < 0.001), carbohydrate (X^2^ = 21.280, *p* < 0.001) and sugar (X^2^ = 8.979, *p* = 0.011). The percentage of products meeting the nutrition claims identified for the bread category are presented in Table 3. In summary, of all bread products, 29% were eligible to make a whole grain claim with 27% of bread products very high in whole grain (≥24 g/serve) (up from 18% in 2014). Within loaf breads (*n* = 243), 40% were at least a source of whole grain (≥8 g/serve), 79% were at least a ‘source’ of dietary fibre, 54% met the Australian Government’s reformulation target of ≤400 mg/100 g sodium, 78% were a source of protein, 20% were a ‘good source’ of protein (10 g/serve), and the majority (97%) were low in sugar.

The mean HSR for white loaves was only ½ a star below that for whole grain varieties (3.7 versus 4.2) and for gluten-free loaves, a mean of 4 stars, possibly due to the lower protein content. The total number of bread loaves with a HSR was 40, representing 28% of the audited products and four products in this audit displayed less than three stars. There was little change in the number of gluten-free products over time, with one less loaf in this audit and an increase in rolls from 2 to 6 products between 2014 and 2017. 

### 3.1. Whole Grain

Whole grain sliced loaves, rolls and sandwich thins were the best source of whole grain within the bread category. Whole grain loaves were higher in fibre, protein and contained less carbohydrate and sugar than other loaf breads. No white rolls, gluten-free rolls or pita breads were eligible to make a whole grain claim. One gluten-free loaf could make a whole grain claim. One white sliced loaf was labeled as whole grain and contained a claimable amount of whole grain, so this product was included in the whole grain bread category. Seventy-nine products made an on-pack claim about whole grain, and these were mostly wholemeal bread loaves. Sixty additional products were eligible but did not make a whole grain claim and 17% of all bread products (*n* = 78) were not assessable for their whole grain content, as they did not provide the percentage of whole grain in the ingredients list. Compared to 2014, there were 20 fewer white loaves and 20 additional whole grain loaves available for purchase.

### 3.2. Dietary Fibre

Whole grain sliced loaves and rolls were the best source of dietary fibre within the total bread category with a median of 4.6 g per serve, or one sixth of the daily 30 g Adequate Intake [32]. The fibre content of white bread was less than half that of whole grain varieties. One product made a fibre claim and was ineligible to do so. No other issues of this type were found through this audit.

### 3.3. Sodium

More than half of all breads (237 products) met the Australian Government’s reformulation target of ≤400 mg/100 g sodium [33]. Sodium content varied widely between 57 to 1400 mg per 100 g in this audit with a median of 400 mg/100 g across all bread types from the total bread category. One bread a German Pumpernickel bread (Delba’s^TM^, Delbrück, Germany) was the outlier with 1400 mg/100 g. Just two products could claim low sodium (<120 mg/100 g). Gluten-free versions had the lowest levels of the loaves audited, with 90% meeting the reformulation target. Compared with the bread audit conducted by GLNC in 2014, the median sodium content for all breads is now 11% lower (450 mg/100 g, *n* = 467).

### 3.4. Protein

One in every five whole grain sliced loaves and 42% of flatbreads were a ‘good source’ of protein, offering at least 10g per serve. Close to a quarter of white sliced loaves were a ‘*source*’ of protein, with at least 5 g per serve. Gluten-free sliced loaves and sandwich thins contained the least protein.

### 3.5. Carbohydrate and Sugar 

Carbohydrate (CHO) ranged from 5 g/serve to 76.4 g/serve in the total bread category with a median of 30 g/serve, typically two slices. Flatbreads contained the most CHO (mostly determined by the larger serving size of 114 g) and Sandwich Thins were the lowest. Within loaves, there was a slight advantage in choosing whole grain varieties which were slightly lower in carbohydrate, with one product containing only 5 g/serve. Nearly all breads captured (90%) were considered low in sugar (≤5 g/100 g). Bakery breakfast products were the exception, and included sweet breads like brioche, pancakes/pikelets and fruit bread, these had an average of 10 g/serve. 

## 4. Discussion

There are important findings from this research to guide product development within industry, and correct some of the misinformation regarding bread, particularly for nutrition professionals assisting Australians to achieve the 48 g DTI for whole grains. Of all bread products examined, one-third were eligible to make a whole grain claim, with 27% of bread products very high in whole grain (≥24 g/serve) and many of these easily providing 100% of the DTI for whole grain. Loaf breads were the most frequently consumed type of bread, so the fact that 79% of loaves were at least a source of fibre and an even higher proportion (40%) were at least a source of whole grain (≥8 g/serve) is reassuring. Importantly, whole grain varieties within the category of loaf breads have increased over time, seemingly replacing white loaves. The reason for manufacturers of 60 eligible products not claiming whole grain content is unclear, and is an opportunity within the current market as this category expands.

In relation to whole grain in bread, despite the uptake by industry, consumption studies suggest that only about one-third of Australians meet the recommended DTI of 48 g [22]. The Australian Dietary Guidelines suggest ‘mostly whole grain’ [34], encouraging consumption, but at the same time respecting the value of core, refined grain foods in diets. However, it is recognised that more specific guidance is needed to achieve higher quality diets for disease prevention [35]. Consumption of whole grain foods was calculated from the NNPAS at 21 g/day for adults (19–85 year) and 17 g/day for children (2–18 year) [22] and a similar level was derived from the most recent consumption research commissioned by GLNC (*n* = 1121) at 26 g/day (9–70 year) and 16 g/day for children [36]. These results are similar to those gathered in the UK [24], USA [23], Ireland [37] and Singapore [38] while other countries, such as France [39], Italy [40] and Malaysia [41] have far lower intakes. Scandinavian countries have recently increased intakes, in particular Denmark, who has successfully promoted whole grains, increasing consumption from 33 g/day (from 2000–2004) to 55 g/day (in 2011–2014) [42]. On average, bread products have been shown to provide 20% of the whole grain Daily Target Intake (DTI), with 40% from cereals and a further 19% from ‘other breads’ like tortillas [36]. 

Bread has been the target of negative press, despite being a vehicle for a range of key nutrients within the Australian diet. This includes nutrients of concern such as dietary fibre, iron and thiamine but also folate and iodine, the latter two being mandatory fortificants added to bread-making flour since 2009. Importantly, following folic acid fortification in Australia, there was a statistically significant 14.4% decrease in the rate of neural tube defects (NTD) across the population (excluding Victoria, the Australian Capital Territory and Tasmania where insufficient data was available) [6]. This reduction was predicted prior to mandating fortification and was based on consumption of three slices of bread per day [6], plus other measures (such as supplements) within at risk groups. The impact of folic acid fortification was even greater among Aboriginal and Torres Strait Islander women with a 72.2% reduction of NTD [6]. 

In the case of iodine, the Australian population is now reportedly consuming sufficient iodine to address the recent re-emergence of mild iodine deficiency at the population level. However, studies in South Australia and Western Australia indicate that pregnant and breastfeeding women may still require supplements and iodised salt [3,43]. In New Zealand there have been more modest improvements and some target groups remain at risk of mild iodine deficiency [6]. For a successful fortification program, consumption of the fortified food needs to remain fairly stable. As less bread is being consumed in Australia, the nutrients from this staple may need to be provided by other foods or drinks [44,45]. Whereas National data indicated a 20 g decline in bread consumption [5], GLNC data show a larger reduction [46], from 124.5 g in 2014 down to 66 g in 2017. A study of cereal foods from Western Australia also found a decline in intake over the same period of time (1995 through to 2012) and concluded there were misperceptions as to the adequacy of intake of grain foods, specifically whole grains [35].

More than half (54%) of all loaves audited met the Australian Government’s reformulation target of ≤400 mg/100 g for sodium and two products were able to claim low sodium. All Lebanese and sandwich thins contained ≤400 mg/100 g, and consumption of these products is increasing [46]. Sodium is a current focus within the public health domain [47] internationally and in Australia, with targets for sodium in bread expected to be reduced to ≤380 mg/100 g as part of the Healthy Food Partnership reformulation proposal. However, reformulation needs to be balanced against the role that bread plays in supplying fortificants like iodine. Salt in bread controls the yeast through fermentation and stabilises gluten [44] and, therefore, impacts on how the dough handles as it moves through modern production lines. Sodium also influences shelf life and the final texture and flavour of the bread [48,49]. However, there is scope for sodium reduction, and in bread, 300 mg/100 g has been tested [47]. The results from this audit are comparable to Trevena et al. [49] who found that mean sodium from approximately 150 bread varieties was 415 mg/100 g in 2013, 9% lower than an audit conducted in 2010, where 67% met the reformulation target. From the GLNC 2014 audit, median sodium content of 450 mg/100 g (*n* = 467) was reduced to 400 mg in 2017. This was an 11% reduction with just over half of bread varieties meeting the current sodium target. Lindberg et al. [50] also showed improvements with 86% meeting the target in 2015, an improvement from 28% at baseline when the sodium target was introduced [51]. 

Close to one in every five loaves of whole grain bread assessed was considered a ‘good source’ of protein, with at least 10 g per serve—the same amount found in a glass of milk or two boiled eggs. Almost three quarters (73%) of white sliced loaves were a ‘source’ of protein, with at least 5 g per serve. Consumers are interested in plant proteins, and they may not consider bread as a potential source. In addition to grains and seeds, ingredients like legumes and legume flours are being added to bread (5% of all breads in this audit) in the form of lentils, lupin bran, broad bean flour, besan flour, mung beans and soy beans in various forms (kibbled and as flour). Although the evidence supports the plant protein trend in terms of health, particularly cardiometabolic health, the literature supports a balanced view which includes amounts of animal protein as per current dietary guidelines [52]. However, plant protein is a platform that is being used by some manufacturers to promote higher protein content via pack messages.

There is consumer concern about the added sugar in bread and this was one of the top four reasons Australians reportedly avoided bread in 2017 [46]. Sugar is added in small amounts of 1–2% to bread to accelerate processes, produce an attractive crust colour and help tenderize bread [53]. In excess, sugar weakens the gluten network by competing for water [54]. From this audit, the majority (89%) of loaves were low in sugar (less than 5 g/100 g), and this figure includes bakery breakfast products which contain dried fruit. When considering sliced loaves alone, 92% were low in sugar. Added sugar in the food supply is a current issue in Australia as labels on packaged foods and drinks do not provide a breakdown of intrinsic and added sugar and this may be a barrier for consumers in making informed choices.

Despite the apparent trend in gluten-free and wheat-free diets over the last several years and the expansion of these products within the mainstream food supply [55,56], there was little difference within the bread category in the number of sliced gluten-free loaves or rolls over time demonstrated by this audit. By excluding in-store bakery and limiting our analysis to loaf breads, some products may have been omitted from the gluten-free category that have been included in analyses by other authors, for example, Wu et al. (2015) [57] who analysed 54 gluten-free bread products. Importantly, there may be a perception among consumers that gluten-free is a healthier choice, but several studies support the opposite conclusion [56,57] with unfavourable changes to the microbiome and no impact on inflammatory markers [58,59]. Lower nutrient intakes, particularly lower protein and whole grain content were apparent from this study and others [56]; however, gluten-free loaves tended to be lower in sodium than white breads, with 90% meeting the reformulation target.

This study benefits from having a perspective of the total bread category, while the analysis focuses on just loaf breads. However, there are limitations as it is difficult to estimate the total bread market with accuracy due to the contribution of small, local bakeries and supermarket in-store bakeries to bread consumption. Specifically, exclusion of in-store bakeries may have limited the number of gluten-free products included compared to other studies [57]. Regarding the data collected, it is not mandatory to report the dietary fibre in the NIP unless there is an on-pack claim. Similarly, reporting whole grain is voluntary, so whole grain content (as a percentage) may not always be declared and as such, there may be missing data. We did not conduct our own analysis of nutrient content and were reliant on manufacturer data, this is an unavoidable limitation. All care was taken to ensure the quality of the data and limit error in tabulation through quality checking by an independent reviewer and packaging photographs assisted with this process.

## 5. Conclusions

There has been a change in the way bread is used in diets. Previously, a common inclusion at each meal of the day, to the current day, where some consumers are actively avoiding bread or reducing consumption. This audit highlights some of the key nutrients in breads, compliance with reformulation targets and the change in the market toward increasing whole grain and high fibre varieties. Continued clear messaging on pack by food industry, combined with consistent messaging from healthcare professionals, government bodies and Dietary Guidelines about the value of bread in the diet pattern is needed. This is crucial if bread is to continue as an effective food vehicle for broad fortification programs. However, greater efforts may be required to change the negative attitudes to carbohydrate foods in general and support promotion of core foods in a positive manner. Communication tools that reflect the value of grain foods and whole grain food choices in particular, are needed to encourage consumers to make better food choices. In this respect, the Code of Practice for Whole Grain Ingredient Content Claims provides a model for regulation and permitted on-pack claims. The potential inclusion of whole grain as part of the Health Star Rating system is also an opportunity that may assist Australians in reaching the 48g whole grain target, with potential positive health outcomes. 

## Figures and Tables

**Table 1:** Nutrition claims and reformulation target relevant for the bread category.

Nutrition Claim	Whole Grain	Fibre	Sodium	Protein	Sugar
**Claim Criteria**	≥8 g WG/serve	At least a source ≥2 g/serve	Low Sodium <120 mg/100g	‘Source’ ≥5 g/serve	Low in Sugar: ≤5 g per 100g
	‘Contains’ 8–16 g WG/serve	Source ≥2–4 g/serve	Reformulation target <400 mg/100 g	‘Good source’ ≥10 g/serve	
	‘High’ 16–24 g WG/serve	Good source ≥4–7 g/serve			
	‘Very high’ ≥24 g WG/serve	Excellent source ≥7 g/serve			

**Table 2:** Whole grain content and key nutrients in loaf breads (white, whole grain and gluten-free) and in the total bread category.

Type	Whole Grain Median (Range) (g per serve)	Dietary Fibre Median (Range) (g per serve)	Sodium Median (Range) (mg per 100 g)	Protein Median ((Range) (g per serve)	Carbohydrate Median (Range) (g per serve)	Sugar Median (Range) (g per serve)
White (*n* = 80)	4.8 (2.5–6)	2.2 (1–7)	479.5 (237–710)	6.0 (1–12)	31.6 (16–57.8)	1.45 (0–7)
Whole Grain (*n* = 141)	20.1 (2–75)	4.6 (1–11)	400.0 (199–1400)	7.5 (2–24)	28.2 (5–60.5)	1.7 (0–8)
Gluten -Free (*n* = 22)	1.6 (1–14)	3.0 (1–7)	400.0(280–500)	4.1 (1–8)	32.1 (11.5–39.1)	3.0 (0–5)
*P* value * (between loaf breads)	*p* = 0.001	*p* < 0.0001	*p* < 0.0001	*p* < 0.0001	*p* < 0.0001	*p* = 0.011
Total bread category (*n* = 456)	18.2 (0–75)	3.0 (0–11)	400.0 (57–1400)	6.1 (1–24)	30.0 (5–76.4)	1.8 (0–30)

* Kruskal-Wallis test (*p* < 0.005) (IBM SPSS^®^, version 25.0, IBM Corp., Chicago, IL, USA).

**Table 3:** Percentage of loaf breads and total bread meeting the claim criteria and sodium reformulation target.

Claim Criteria	White (*n* = 80)	Whole Grain (*n* = 141)	Gluten-Free (*n* = 22)	Total Bread (*n* = 456)
Eligible for WG claim	0	67	5	28
Contains WG	0	27	0	25
High in WG	0	21	0	22
Very high in WG	0	52	0	51
At least a source of fibre	62	89	81	68
Source of fibre	47	32	48	54
Good Source of fibre	15	47	24	37
Excellent Source of fibre	0	10	9	8
Low in Sodium	0	0	0	5
Reformulation Target <400 mg/100 g	37	58	90	52
Source of protein	73	65	24	57
Good source of protein	7	20	0	12
Low in sugar	97	89	90	90
